# Conservative fluid therapy in septic shock: an example of targeted therapeutic minimization

**DOI:** 10.1186/s13054-014-0481-5

**Published:** 2014-08-29

**Authors:** Catherine Chen, Marin H Kollef

**Affiliations:** Division of Pulmonary and Critical Care Medicine, Washington University School of Medicine, 660 South Euclid Avenue, St Louis, MO 63110 USA

## Abstract

Intravenous fluids (IVFs) represent a basic therapeutic intervention utilized in septic shock. Unfortunately, the optimal method for administering IVFs to maximize patient outcomes is unknown. A meta-analysis of four randomized trials of goal-directed therapy did not demonstrate a significant reduction in mortality (odds ratio 0.609; 95% confidence interval 0.363 to 1.020; *P* = 0.059), whereas 18 trials with historical controls showed a significant increase in survival (odds ratio 0.580; 95% confidence interval 0.501 to 0.672; *P* < 0.0001). Based on these data, clinicians should be aware of the potential for harm due to the excessive administration of IVFs to patients with septic shock.

## Introduction

Intravenous fluids (IVFs) are one of the most common therapies provided to critically ill patients. IVF administration is largely empiric, although goal-directed approaches have been used in an attempt to optimize resuscitation in unstable patients [1,2]. Excessive use of any therapeutic agent can be associated with potential harm; excessive use of antibiotics [3,4], sedation [5,6], tidal volume [7,8], transfusions [9], and glucose [10] have all been linked to unfavorable patient outcomes. It is now recognized that excessive IVFs may also contribute to new complications and worsening of underlying disease processes, including acute respiratory distress syndrome, abdominal compartment syndrome, coagulopathy, and cerebral edema [11-14]. Unfortunately, a systematic approach for delivery of IVFs in critically ill patients does not exist. This is partly related to the various conditions managed in the ICU setting, as well as the varied approaches to IVF administration and availability of different IVF types (that is, colloids, and balanced and unbalanced crystalloids). Goal-directed therapy (GDT) for IVF administration in the first 6 hours of septic shock is now advocated by the most recent version of the Surviving Sepsis Campaign guidelines with a 1C evidence recommendation [1]. However, not all clinicians and investigators are convinced that this approach is optimal [15,16]. Therefore, the goal of this review and meta-analysis is to assess the evidence in support of IVFs in septic shock, focusing on the quantity of IVFs administered, and to determine if conservative fluid therapy is justifiable in septic patients. Like many other therapeutic areas in critical care, an approach to fluid administration that employs therapeutic minimization may be preferred for the avoidance of complications and optimization of patient outcomes.

## Methods

**Figure 1:**
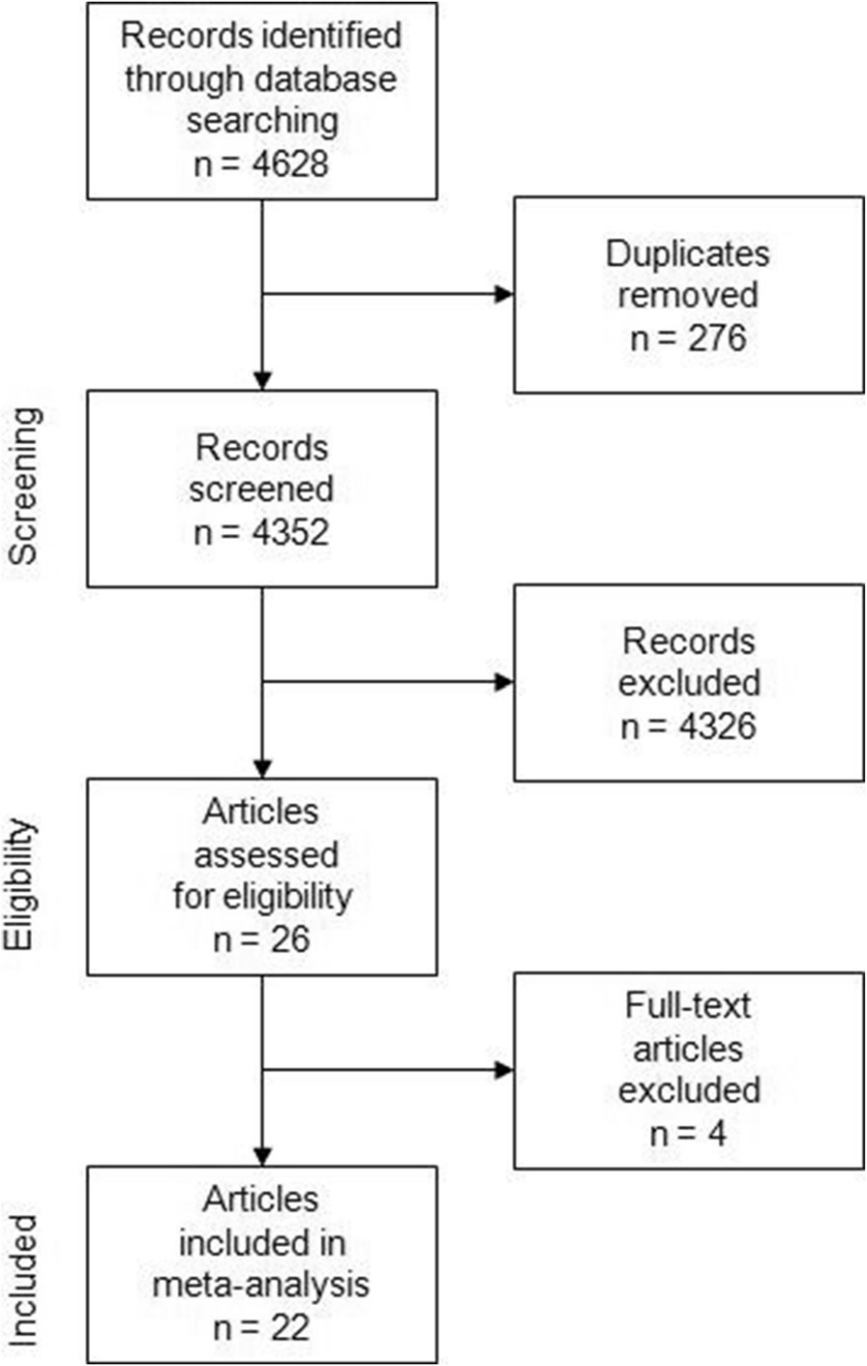
**Summary of evidence search and selection.**

We conducted an English language search of PubMed and Cochrane databases from January 1980 to December 2014 to find human trials of sepsis care bundles in adults (aged ≥18 years) using these search terms: sepsis, septic shock, treatment, guidelines, protocols, GDT, and bundles (Figure 1). The studies that were included had to enroll septic patients, have a control (historical or concurrent), and record mortality rates. The included studies also had to provide targets for their usage of fluids as part of their sepsis intervention or sepsis bundle. Criteria for sepsis or septic shock in patients receiving bundled care had to be consistent with the American College of Chest Physicians and Society of Critical Care Medicine Consensus Conference definitions [17]. Both investigators independently reviewed the included studies by using a standardized data collection form. Discrepancies were resolved by discussion. The Scottish Intercollegiate Guidelines Network checklist for randomized, controlled trials [18] was used to evaluate the methodological quality of the identified studies included in this analysis. A double plus (++) denotes studies very unlikely to have bias, plus (+) studies where bias is unlikely, and minus (−) studies with high risk of bias [18]. Survival was the outcome of interest and tabulated across studies. Conventional forest plots were prepared for survival. A statistical difference between groups was considered to occur if the pooled 95% confidence interval (CI) did not include 1 for the odds ratio (OR). An OR <1 favored bundled GDT when compared with a control group. Two-sided *P*-values were calculated. A random-effects model was chosen for all analyses. Statistical heterogeneity and inconsistency were assessed by using the *Q* and *I*^2^ tests, respectively [19,20]. When the *P*-value of the *Q*-test was <0.10, the *I*^2^ was >25%, or both, heterogeneity and inconsistency were considered significant [19,21].

## Randomized controlled trials

The authors of the Surviving Sepsis Campaign recommend the protocolized, quantitative resuscitation of patients with sepsis-induced tissue hypoperfusion (defined as hypotension persisting after initial fluid challenge or blood lactate concentration ≥4 mmol/L) [1]. They recommend initiating this protocol as soon as hypoperfusion is recognized and it should not be delayed pending ICU admission. During the first 6 hours of resuscitation, the recommended resuscitation goals include all of the following: central venous pressure (CVP) 8 to 12 mmHg; mean arterial pressure ≥65 mmHg; urine output ≥0.5 ml kg h^−1^; superior vena cava oxygen saturation (ScvO_2_) or mixed venous oxygen saturation 70 or 65%, respectively. The primary rationale for this recommendation comes from a randomized, controlled, single-center study demonstrating that early quantitative resuscitation improved survival for emergency department patients presenting with septic shock [2]. Resuscitation targeting the physiologic goals noted above for the initial 6-hour period was associated with a 15.9% absolute reduction in 28-day mortality (46.5% versus 30.5%, *P* = 0.009).

Bundled GDT was also evaluated in a multicenter trial of 314 patients with severe sepsis in eight Chinese centers [22]. This trial reported a 17.7% absolute reduction in 28-day mortality (42.5% versus 24.8%, *P* = 0.001). A single-center randomized controlled study from Taiwan in 224 medical ICU patients using the protocol of Rivers and colleagues [2] also demonstrated a survival advantage with bundled GDT (hospital mortality, 46.3% versus 28.4%, *P* = 0.006) [23]. Most recently, the multicenter ProCESS trial enrolled 1,341 patients into three treatment groups (protocol-based GDT, protocol-based standard therapy, usual care) [24]. No difference in 60-day mortality was observed between the three groups (protocol-based GDT versus usual care: 21.0% versus 18.9%, *P* = 0.830).

**Figure 2:**
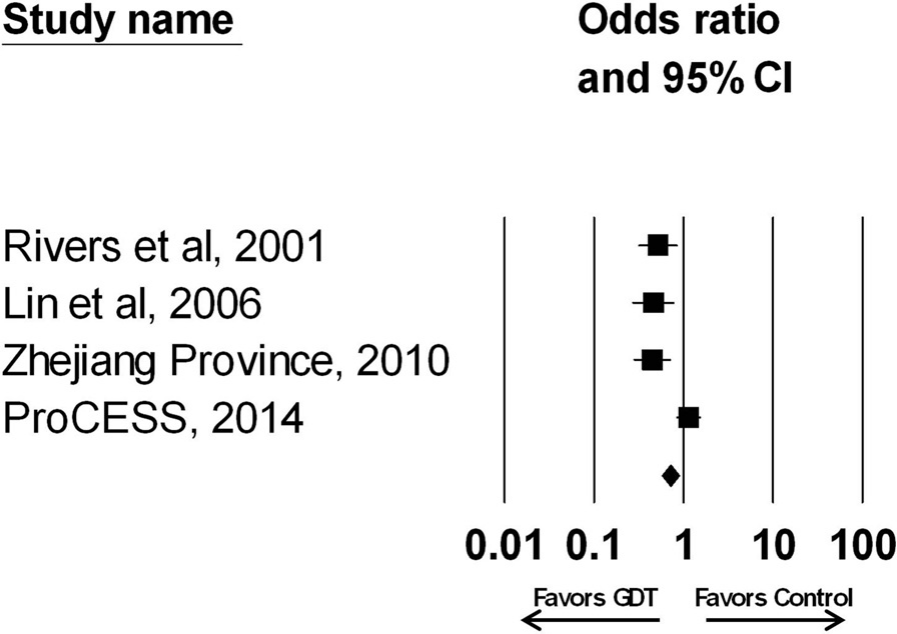
**Effect of bundled goal-directed therapy (GDT) on the odds ratio of survival (95%** **confidence interval (CI)) for the four randomized trials analyzed (odds ratio 0.609; 95%** **CI 0.363 to 1.020;** ***P*** **= 0.059;** ***I*** ^**2**^ **= 80%** **,** ***P*** **= 0.002).** Rivers *et al*. 2001 [2]; Lin *et al*. 2006 [23]; Zhejiang province 2010 [22]; ProCESS 2014 [24].

These four randomized trials included a total of 2,131 patients; 834 (39.1%) in the bundled GDT arm and 851 (39.9%) in the control arm (446 (20.9%) were in the protocol-based standard therapy group of the ProCESS trial). All four studies used the same targeted goals for fluid administration and for the use of vasopressors. The reported total IVF administration for shock varied from 5.0 liters in the control arm of the Taiwanese study to 3.5 liters in the control arm of the Rivers study at 6 hours and 2.3 liters at 6 hours in the control arm of the ProCESS trial (Table 1). Bundled GDT was applied in the emergency department in two studies [2,24] and the ICU in two studies [22,23]. Patients who were treated with bundled GDT did not achieve a significant reduction in mortality compared with those in the control arm (OR 0.609; 95% CI 0.363 to 1.020; *P* = 0.059; Figure 2). The trials were inconsistent and heterogeneous (*I*^2^ = 80%, *P* = 0.002). With removal of the ProCESS trial, the remaining three trials were without heterogeneity and were consistent (*I*^2^ = 0%, *P* = 0.915), demonstrating a significant reduction in mortality with GDT (OR 0.475; 95% CI 0.353 to 0.639; *P* < 0.001). However, these three trials combined had fewer patients enrolled compared to the ProCESS trial.

**Table 1:** **Randomized controlled trials of bundled goal-directed therapy**

**Study and country**	**Sign score**	**Goal-directed therapy (goals)**		**Time and fluid quantification (L)**	
				**Control**	**GDT**
Rivers *et al*. 2001, USA [2]	+	500 ml crystalloid bolus every 30 minutes (CVP 8–12 mmHg)	6 h:	3.5 ± 2.4	5.0 ± 3.0
			7–72 h:	10.6 ± 6.2	8.6 ± 5.2
Lin *et al*. 2006, Taiwan [23]	+	500 ml crystalloid bolus every 30 minutes (CVP 8–12 mmHg)	Total:	5.0 ± 2.9	5.2 ± 4.0
GDT Collaborative Group of Zhejiang Province 2010, China [22]	-	500 ml crystalloid bolus every 30 minutes (CVP 8–12 mmHg)		Quantified but not reported	
ProCESS 2014, USA [24]	++	500 ml crystalloid bolus every 30 minutes (CVP 8–12 mmHg)	6 h:	2.3 ± 1.9	2.8 ± 2.0

Double plus signs (++) indicate studies with very unlikely bias, a single plus sign (+) indicates studies with unlikely bias, and a minus sign (−) indicates studies with high risk of bias. CVP, central venous pressure; GDT, goal-directed therapy.

## Observational trials

**Figure 3:**
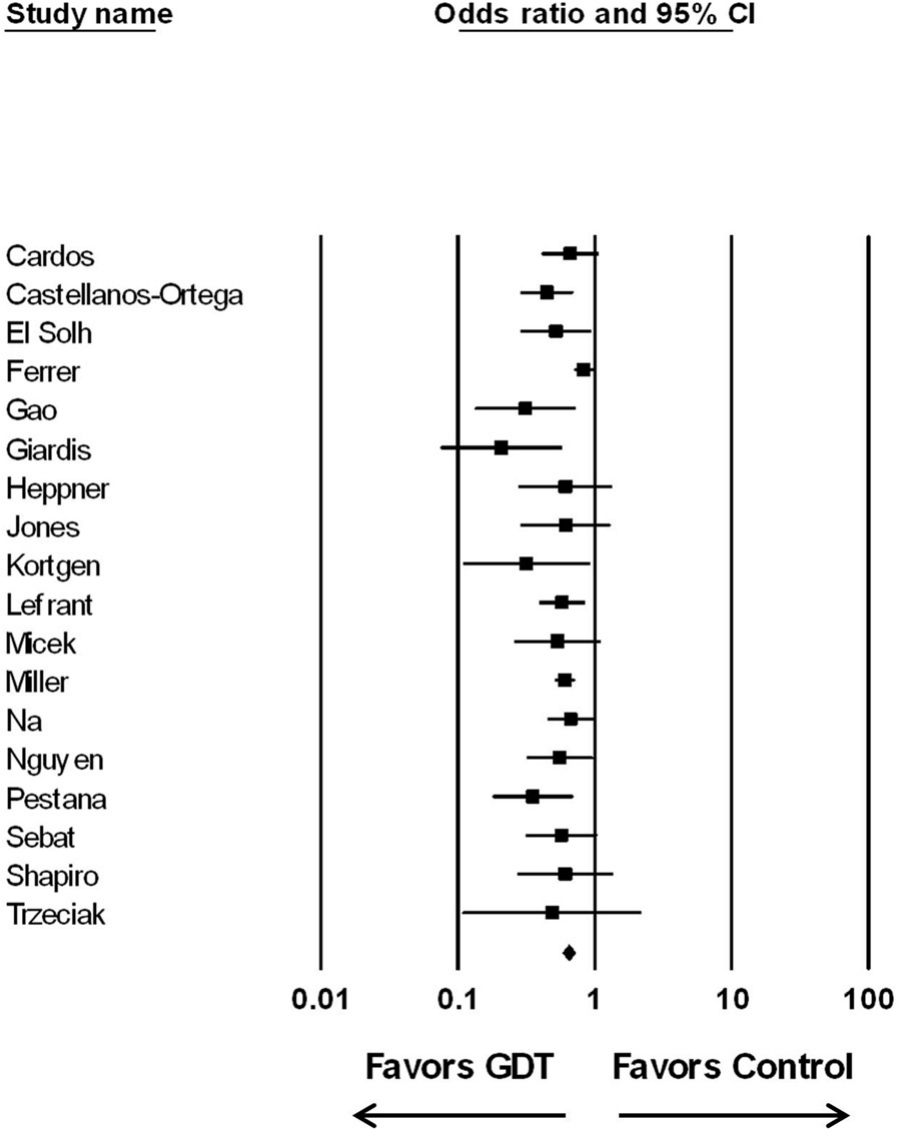
**Effect of bundled goal-directed therapy (GDT) on the odds ratio of survival (95%** **confidence interval (CI)) for the 18 observational trials analyzed.** All 18 studies: odds ratio, 0.580; 95% CI 0.501 to 0.672; *P* < 0.0001 (*I* ^2^ = 32%, *P* = 0.091). With the Ferrer study [33] removed: odds ratio 0.561; 95% CI 0.499 to 0.631; *P* < 0.0001 (*I* ^2^ = 0%, *P* = 0.796).

Eighteen observational trials of bundled GDT were identified [25-42] (Table 2). Seven of these trials were assessed in an earlier analysis demonstrating that a bundled care protocol for septic shock that included the elements of GDT was associated with a significant improvement in hospital survival [15]. Across the 18 studies, the effect of bundled care on survival was heterogeneous and inconsistent (*I*^2^ = 32%, *P* = 0.091; Figure 3). However, when the study by Ferrer and colleagues [33] was removed, the remaining 17 studies were without heterogeneity and were consistent (*I*^2^ = 0%, *P* = 0.796). Overall, there was a statistically significant increase in the odds of surviving with bundled care compared with controls when all studies were examined (OR 0.580; 95% CI 0.501 to 0.672; *P* < 0.0001). Statistical significance was maintained when the Ferrer study was removed from the analysis (OR 0.561; 95% CI 0.499 to 0.631; *P* < 0.0001).

**Table 2:** **Observational controlled trials of bundled goal-directed therapy**

**Study and country**	**Sign score**	**Goal-directed therapy (goals)**		**Time and fluid quantification (L)** ^**a**^	
				**Control**	**Bundled GDT**
Cardoso *et al*. 2010, Portugal [42]	(−)	500 ml to 1000 ml crystalloid bolus, or 300 ml to 500 ml colloid bolus to achieve CVP ≥12 mmHg		Quantified but not reported.	
Castellanos-Ortega *et al*. 2010, Spain [35]	(−)	500-1000 ml crystalloid bolus, additional fluid to achieve CVP ≥8 mmHg		Quantified but not reported.	
El Solh *et al*. 2008, USA [31]	(−)	500 ml crystalloid bolus, repeat until CVP 8–12 mmHg	6 h:	2.5 ± 1.0	3.9 ± 2.0
			24 h:	3.2 ± 1.3	4.9 ± 2.5
Ferrer *et al*. 2008, Spain [33]	(−)	500 ml crystalloid bolus, repeat until CVP 8–12 mmHg		Quantified but not reported	
Gao *et al*. 2005, UK [36]	(−)	Immediate fluid bolus of 0.5 L		Quantified but not reported	
Girardis *et al*. 2009, Italy [34]	(−)	Fluids targeting CVP >6 mmHg or a global end-diastolic volume by trans-pulmonary thermodilution >700 ml/m^2^		Quantified but not reported	
Heppner *et al*. 2012, Germany [39]	(−)	500 ml crystalloid bolus, repeat until CVP 8–12 mmHg		Quantified but not reported.	
Jones *et al*. 2007, USA [30]	(−)	500 ml crystalloid bolus, repeat until CVP 8–12 mmHg	6 h:	2.5 ± 2.4	4.7 ± 1.8
Kortgen *et al*. 2006, Germany [27]	(−)	CVP 8–12 mmHg or intrathoracic blood volume index 850–1000 ml/m^2^	6 h:	2.8 [1.8,3.8]	2.5 [1.6,3.9]
Lefrant *et al*. 2010, France [41]	(−)	≥20 ml/kg crystalloids or colloids within 6 hours	6 h crystalloid:	1.0 [0.5,2.0]	1.5 [0.5,2.0]
			6 h colloid:	0.5 [0.5,1.0]	1.0 [0.5,1.1]
Micek *et al*. 2006, USA [25]	(−)	500 ml crystalloid bolus, repeat until CVP 8–12 mmHg	In ED:	2.8 ± 1.6	3.8 ± 1.7
Miller *et al*. 2013, USA [38]	(−)	500 ml crystalloid bolus, repeat until CVP 8–12 mmHg		Quantified but not reported.	
Na *et al*. 2012, Asia [40]^b^	(−)	Fluid bolus to achieve CVP >8 mmHg by 6 hours	In ED:	1.5 [1.0,2.5]	1.5 [0.9,2.7]
			In ICU:	7.8 [5.2,11.6]	5.6 [3.3,9.2]
Nguyen *et al*. 2007, USA [29]	(−)	CVP ≥8 mmHg	In ED:	2.8 ± 1.5	2.8 ± 2.1
			72 h:	7.8 ± 5.2	7.9 ± 6.1
Pestaña *et al*. 2010, Spain [37]	(−)	Crystalloid within 6 hours to achieve CVP ≥8 mmHg or global end-diastolic volume index ≥680 ml/m^2^		Quantified but not reported.	
Sebat *et al*. 2005, USA [32]	(−)	1000 ml crystalloid in ED, 600 ml increments per MAP & UO protocol		Quantified but not reported.	
Shapiro *et al*. 2006, USA [28]	(−)	500 ml crystalloid bolus, repeat until CVP 8–12 mmHg	6 h:	2.9 ± 1.8	4.1 ± 2.6
			24 h:	6.5 ± 4.5	7.6 ± 3.9
Trzeciak *et al*. 2006, USA [26]	(−)	250-1000 ml crystalloid bolus until CVP ≥8 mmHg	ED:	3.5 ± 2.3	5.7 ± 3.0
			ICU 24 h:	5.5 ± 4.9	2.8 ± 1.7
			ED and ICU 24 h:	9.1 ± 5.1	7.9 ± 3.4

Double plus signs (++) indicate studies with very unlikely bias, a plus sign (+) indicates studies with unlikely bias, and a minus sign (−) indicates studies with high risk of bias. ^a^Values expressed as mean ± standard deviation or median [interquartile range]. ^b^China, India, Taiwan, Singapore, Korea. CVP, central venous pressure; ED, emergency department; GDT, goal-directed therapy; MAP, mean arterial pressure; UO, urine output.

## Limitations of the bundled goal-directed therapy studies

One of the proposed main limitations of the pivotal Rivers trial is the lack of generalizability of the study. For example, Ho and colleagues [43] examined 4,784 emergency department patients and found only 40 (0.8%) were candidates for bundled GDT. Moreover, these investigators found patient mortality to be 26.0%, much lower than the mortality observed in the control arm of the Rivers study, which was 46.5%, and the control arm mortality in the other two older randomized trials of bundled GDT [2,22,23,43]. Variability in mortality was also observed in the 18 observational studies examined where the mortality of the control arms ranged from 15.5% to 67.6% [25-42]. Interestingly, the ProCESS trial had one of the lowest observed mortality rates in their control arm at 18.9%. Other limitations of these trials include resuscitation protocol complexity, potential risks associated with elements of the protocol (especially the use of dobutamine and red blood cell transfusions), and financial and infrastructure implications necessary to carry out these protocols. For example, in the Rivers trial the bundled GDT patients had a dedicated investigator to ensure compliance with the study protocol and had central venous catheters placed to continuously assess ScvO_2_.

The observational studies of bundled GDT have a number of other important limitations, foremost being their lack of scientific rigor (Table 2). It is also important to note that the earlier meta-analysis of seven of these trials found that only time to antibiotic administration (in hours) between bundle and control patients was consistent between studies, whereas crystalloids, vasopressors, inotropes, packed red blood cell transfusion, corticosteroids, and drotrecogin alfa (activated) exhibited significant heterogeneity between the studies [15]. The overall importance of timely antibiotic therapy was further supported by the analysis of the Spanish study that examined 2,319 patients showing a reduction in mortality with bundled GDT [33]. In a subsequent analysis of their data, the Spanish investigators reported on compliance with four therapeutic goals and four treatments employed in their bundle [44]. Only timely administration of antibiotics and drotrecogin alfa (activated) for multiorgan failure were associated with significantly lower mortality [44].

**Figure 4:**
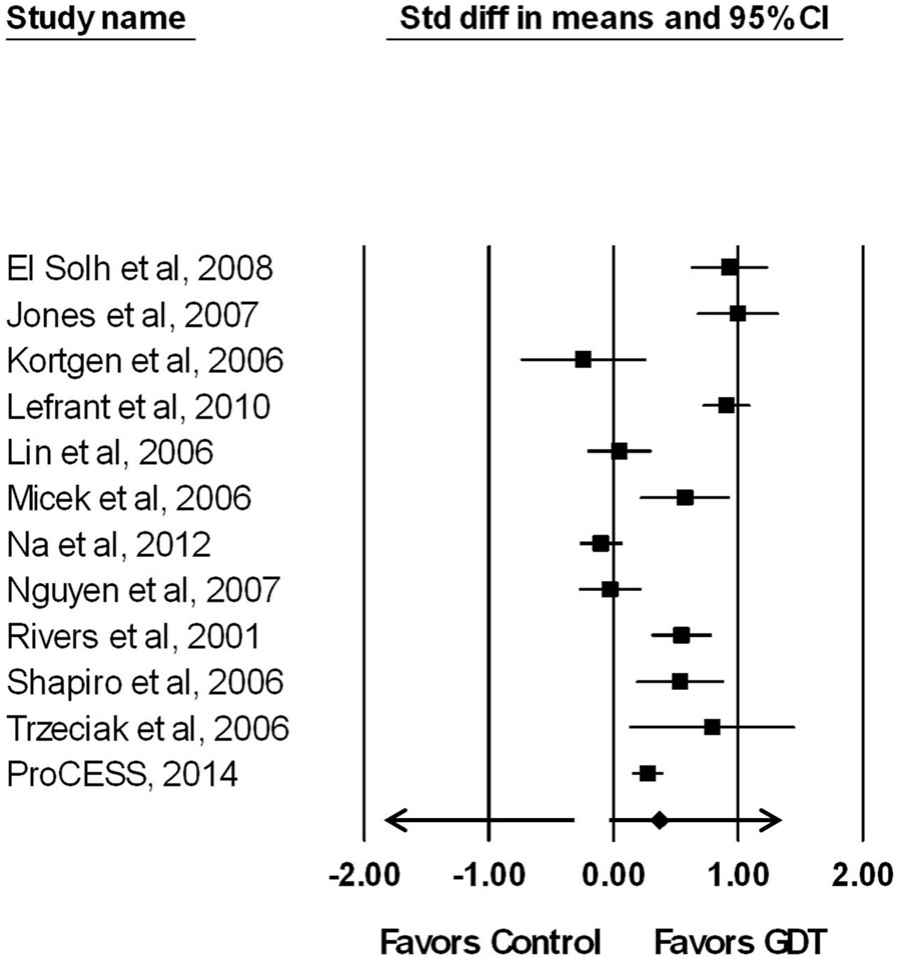
**Effect of bundled goal-directed therapy (GDT) on the standardized paired difference (Std diff) of the means for intravenous fluid use based on study defined resuscitation goals (** ***I*** ^**2**^ **= 90%** **,** ***P*** **< 0.001).** CI, confidence interval.

Another important limitation of both the randomized trials and the observational studies examining bundled GDT is that multiple interventions occurred that could potentially influence patient outcome. This is supported by the observation that statistical differences in the use of vasopressors, red blood cell transfusions, corticosteroids, and timely administration of antibiotics existed between study arms when the trials reporting specific interventions were combined for analysis (Table 3). Moreover, across the 12 studies reporting on IVF administration, most showed greater fluids administered to patients receiving bundled GDT (median (interquartile range): 3,875 ml (2,638 ml, 4,901 ml) versus 2,779 ml (2,332 ml, 3,342 ml); *P* = 0.143) (Tables 1 and 2). However, the studies were inconsistent and significant heterogeneity existed between studies for the difference in IVFs administered by treatment group (*I*^2^ = 90%, *P* < 0.001) (Figure 4). Removal of any one study failed to significantly reduce heterogeneity (*I*^2^ remained 87% to 91%, with *P* < 0.001).

**Table 3:** **Comparison of specific interventions employed in trials of bundled goal-directed therapy**

	**Vasopressors**		**Inotropes**		**PRBC**		**Corticosteroids**		**rhAPC**		**Appropriate antibiotics**		**Timely antibiotics**	
**Study**	**Control**	**Bundled care**	**Control**	**Bundled care**	**Control**	**Bundled care**	**Control**	**Bundled care**	**Control**	**Bundled care**	**Control**	**Bundled care**	**Control**	**Bundled care**
ProCESS [24]	201/456	241/439	4/456	35/439	34/456	63/439	37/456	54/439	0/456	1/439	442/456	428/439	NA	NA
Rivers *et al*. [2]	40/133	36/130	1/133	18/130	25/133	83/130	NA	NA	NA	NA	125/133	126/130	123/133	112/130
Lin *et al*. [23]	81/116	80/108	16/116	13/108	43/116	39/108	25/116	32/108	NA	NA	107/116	102/108	NA	NA
El Solh *et al*. [31]	NA	NA	NA	NA	11/87	12/87	14/87	83/87	2/87	11/87	73/87	84/87	79/87	83/87
Ferrer *et al*. [33]	329/854	630/1,465	NA	NA	NA	NA	311/854	611/1,465	51/854	74/1,465	568/854	1,009/1,465	NA	NA
Jones *et al*. [30]	27/79	53/77	1/79	2/77	1/79	4/77	5/79	31/77	3/79	3/77	NA	NA	NA	NA
Kortgen *et al*. [27]	NA	NA	0/30	6/30	5/30	5/30	13/30	30/30	0/30	7/30	28/30	28/30	30/30	30/30
Lefrant *et al*. [41]	NA	NA	40/230	20/215	24/230	34/215	91/230	122/215	0/230	4/215	NA	NA	141/230	145/215
Micek *et al*. [25]	60/60	43/60	NA	NA	4/60	12/60	30/60	13/60	7/60	2/60	43/60	52/60	36/60	52/60
Na *et al*. [40]	171/364	135/192	144/364	78/192	17/364	4/192	NA	NA	NA	NA	NA	NA	NA	NA
Nguyen *et al*. [29]	39/77	111/253	18/77	67/253	11/77	32/253	23/77	41/253	6/77	4/253	NA	NA	77/77	227/253
Shapiro *et al*. [28]	23/51	63/79	1/51	6/79	3/51	8/79	12/51	23/79	0/51	3/79	45/51	77/79	48/51	78/79
Trezeciak *et al*. [26]	7/16	13/22	0/16	2/22	0/16	3/22	5/16	8/22	2/16	7/22	NA	NA	NA	NA
Totals	978/2206 (44.3)	1,405/2,825 (49.7)	225/1,552 (14.5)	247/1,545 (16.0)	178/1,699 (10.5)	299/1,692 (17.7)	566/2,056 (27.7)	1,048/2,835 (37.0)	71/1940 (3.7)	116/ 2,727 (4.3)	1,431/1,787 (80.1)	1,906/2,398 (79.5)	534/668 (79.9)	727/854 (85.1)
*P*-values^a^	<0.001		0.249		<0.001		<0.001		0.308		0.636		0.008	

^a^ *P*-values for comparison between control and bundled care groups for each intervention. NA, not available; PRBC, packed red blood cells; rhAPC, recombinant human activated protein C.

For completeness, one additional observational study was identified but not included in our analysis due to its makeup. This was a multicenter quality improvement study conducted in the United States, Europe, and South America utilizing a multifaceted intervention to facilitate compliance with selected guideline recommendations for the management of severe sepsis [45]. This intervention included the use of a CVP greater than 8 mmHg to target fluid administration. Data on 15,022 patients at 165 hospitals were included in the analysis. Compliance with the initial 6-hour bundle targets increased linearly from 10.9% of subjects in the first quarter to 31.3% by the end of 2 years of the quality improvement campaign. This was associated with a significant reduction in mortality over the same time period (37.0% in the first quarter in the campaign to 30.8% by 2 years, *P* = 0.001). After adjustment for baseline characteristics, administration of broad-spectrum antibiotics, obtaining blood cultures before antibiotic initiation, administration of drotrecogin alfa (activated) in the first 24 hours, achieving plateau pressure control, and maintaining blood glucose control were all associated with lower hospital mortality. In those with septic shock, there was no association between mortality and the ability to achieve a CVP ≥8 mmHg or demonstration of ScvO_2_ ≥ 70% [45].

The limitations of the available clinical studies of bundled GDT, to include the inconsistent results regarding IVF administration, have served as the major impetus for the conduct of three multicenter trials examining the elements of GDT (ARISE in Australasia, ProMISe in the United Kingdom, and ProCESS in the United States) [24,43,46]. These trials have similar structures, interventions, and patient entry criteria that will allow the trial investigators to collaboratively conduct a prospective individual patient data meta-analysis, using the raw data from each trial [46]. With over 4,000 subjects combined, these trials are powered to find smaller effects on outcome and to better explore subgroups. The ProCESS trial has already been published and showed that protocolized GDT did not reduce mortality compared with usual care [24]. However, patients in the usual care arm received significantly less IVF at 6 hours and had a numerically lower mortality compared to the protocolized GDT arm.

## Conclusion

Clinicians caring for critically ill patients must always weigh the benefits and risks of administered therapies to include the use of IVFs. Our meta-analysis supports the findings of an earlier analysis [15] demonstrating that IVF volume was not consistently altered by the use of GDT bundles, and thus firm recommendations regarding their quantitative use cannot be made. Clinicians should at least be aware of the potential for harm due to the excessive administration of IVFs to patients with septic shock.

## Abbreviations

CI: Confidence interval
CVP: Central venous pressure
GDT: Goal-directed therapy
IVF: Intravenous fluid
OR: Odds ratio
ScvO_2_: Superior vena cava oxygen saturation
